# Supplementary material for: The SUN1-SPDYA interaction plays an essential role in meiosis prophase I
Source: Nat Commun. 2021 May 26;12:3176. doi: 10.1038/s41467-021-23550-w (PMC8155084; doi:10.1038/s41467-021-23550-w)
Supplement: Supplementary file 1 — Supplementary Information [file 41467_2021_23550_MOESM1_ESM.pdf]

Supplementary Information

The SUN1-SPDYA interaction plays an essential role in meiosis prophase I

Y Chen, Y Wang et al

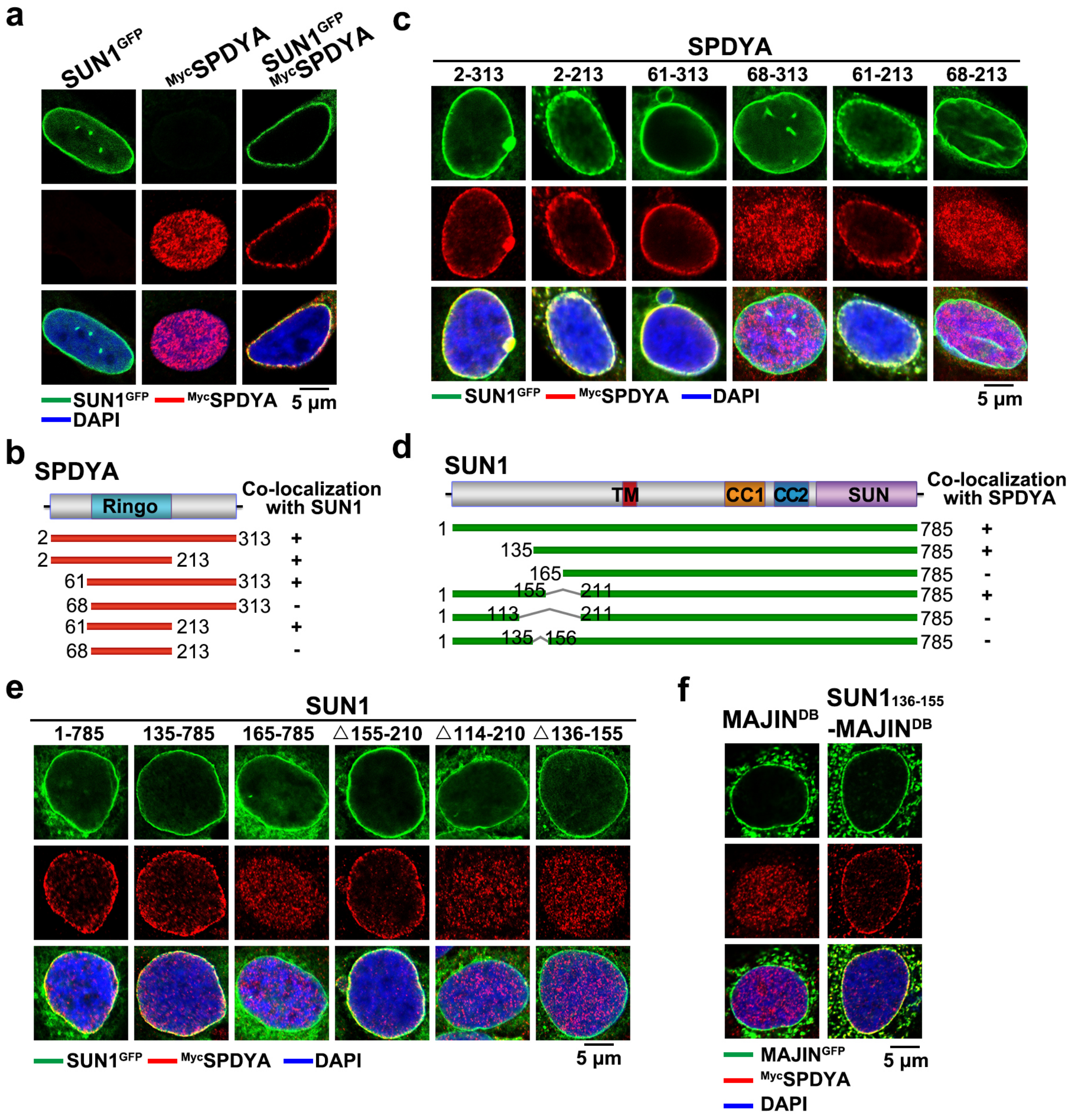
a
SUN1GFP
MycSPDYA
SUN1GFP MycSPDYA
SUN1GFP MycSPDYA DAPI
5 μm
b
SPDYA
Ringo
Co-localization with SUN1
2 313 +
2 213 +
61 313 +
68 313 -
61 213 +
68 213 -
c
SPDYA
2-313
2-213
61-313
68-313
61-213
68-213
SUN1GFP MycSPDYA DAPI
5 μm
d
SUN1
TM
CC1
CC2
SUN
Co-localization with SPDYA
1 785 +
135 785 +
165 785 -
1 155 211 785 +
1 113 211 785 -
1 135 156 785 -
e
SUN1
1-785
135-785
165-785
△155-210
△114-210
△136-155
SUN1GFP MycSPDYA DAPI
5 μm
f
MAJINDB
SUN1136-155 -MAJINDB
MAJINGFP
MycSPDYA
DAPI
5 μm

Supplementary Figure 1: Characterization of the interaction between SUN1 and SPDYA.

a, Equator images of U-2 OS cells expressing SUN1-GFP (green) and/or Myc-SPDYA. Cells were immunostained with anti-Myc antibody (red). DNA was stained by DAPI (blue). Scale bar, 5 μm. **b**, Co-localization analysis of ectopically expressed SUN1-GFP and various Myc-SPDYA fragments. **c**, Representative IF images for panel **b**. U-2 OS cells expressing SUN1-GFP (green) and Myc-SPDYA were immunostained with anti-Myc antibody (red). Scale bar, 5 μm. **d**, Co-localization analysis of ectopically expressed Myc-SPDYA and various SUN1-GFP fragments. **e**, Representative IF images for panel **d**. U-2 OS cells expressing SUN1-GFP (green) and Myc-SPDYA were stained with anti-Myc antibody (red). TM: trans-membrane; CC: coiled-coil domains. Scale bar, 5 μm. **f**, Equator images of U-2 OS cells expressing $MAJIN^{DB}$-GFP or $SUN1_{136\text{-}155}$-$MAJIN^{DB}$-GFP (green) together with Myc-SPDYA (red). Ectopically expressed DNA-binding defective mutant MAJIN ($MAJIN^{DB}$) exhibited clear nuclear membrane localization[13]. Scale bar, 5 μm.

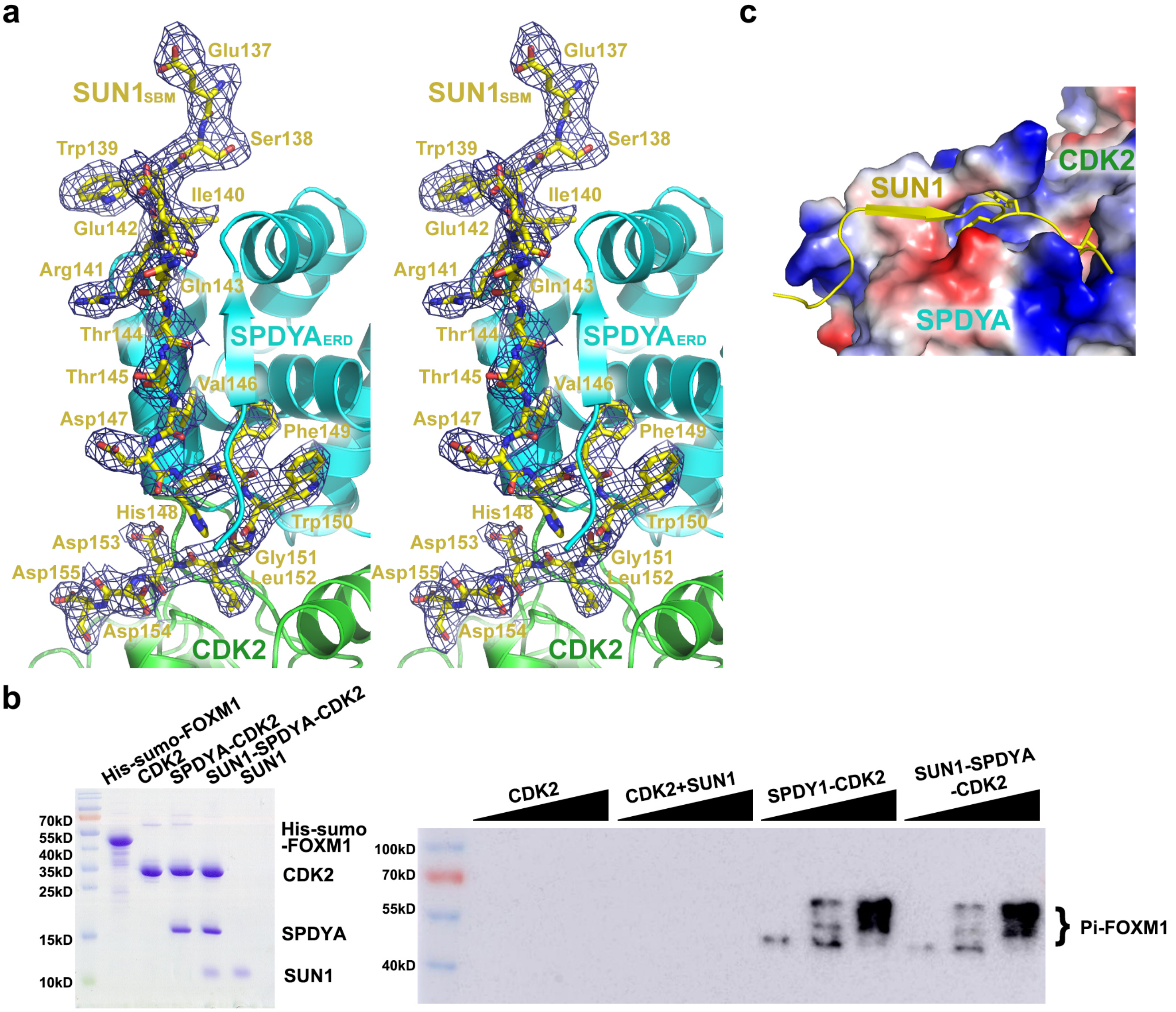

a
SUN1SBM
Glu137
Ser138
Trp139
Ile140
Glu142
Arg141
Gln143
Thr144
Thr145
Val146
Asp147
Phe149
His148
Trp150
Asp153
Gly151
Asp155
Leu152
Asp154
SPDYAERD
CDK2
c
SUN1
CDK2
SPDYA
b
His-sumo-FOXM1
CDK2
SPDYA-CDK2
SUN1-SPDYA-CDK2
SUN1
70kD
55kD
40kD
35kD
25kD
15kD
10kD
His-sumo
-FOXM1
CDK2
SPDYA
SUN1
CDK2
CDK2+SUN1
SPDY1-CDK2
SUN1-SPDYA
-CDK2
100kD
70kD
55kD
40kD
Pi-FOXM1

Supplementary Figure 2: Structural and phosphorylation analyses of the $SUN1_{SBM}$-$SPDYA_{ERD}$-CDK2 complex.

a, Stereo view of the $SUN1_{SBM}$-$SPDYA_{ERD}$-CDK2 complex focused on the $SUN1_{SBM}$ polypeptide. The electron density (2Fo-Fc) map of SUN_{SBM} contoured at 1.0 σ is shown in the complex. **b**, Phosphorylation analysis of His-Sumo-$FoxM1_{526\text{-}748}$ by the indicated protein or protein complexes. His-Sumo-$FoxM1_{526\text{-}748}$, $SUN1_{131\text{-}160}$, CDK2 and CDK2 complexes were expressed in bacteria and purified by affinity chromatograph. A Coomassie blue-stained gel showed the purified recombinant proteins (left). *In vitro* kinase assay was performed by incubating CDK2 or CDK2 complex as indicated at three different concentrations (0.03 μM, 0.1 μM, 0.3 μM), together with 1 μM FoxM1 substrate at 30 °C for 20 min. Phosphorylation of FoxM1 was detected by western blotting using anti-phospho-Thr-Pro antibody (right). **c**, Electrostatic surface potential of $SUN1_{SBM}$ binding site in the $SPDYA_{ERD}$-CDK2 complex. Positive potential, blue; negative potential, red. Source data are provided as a Source Data file.

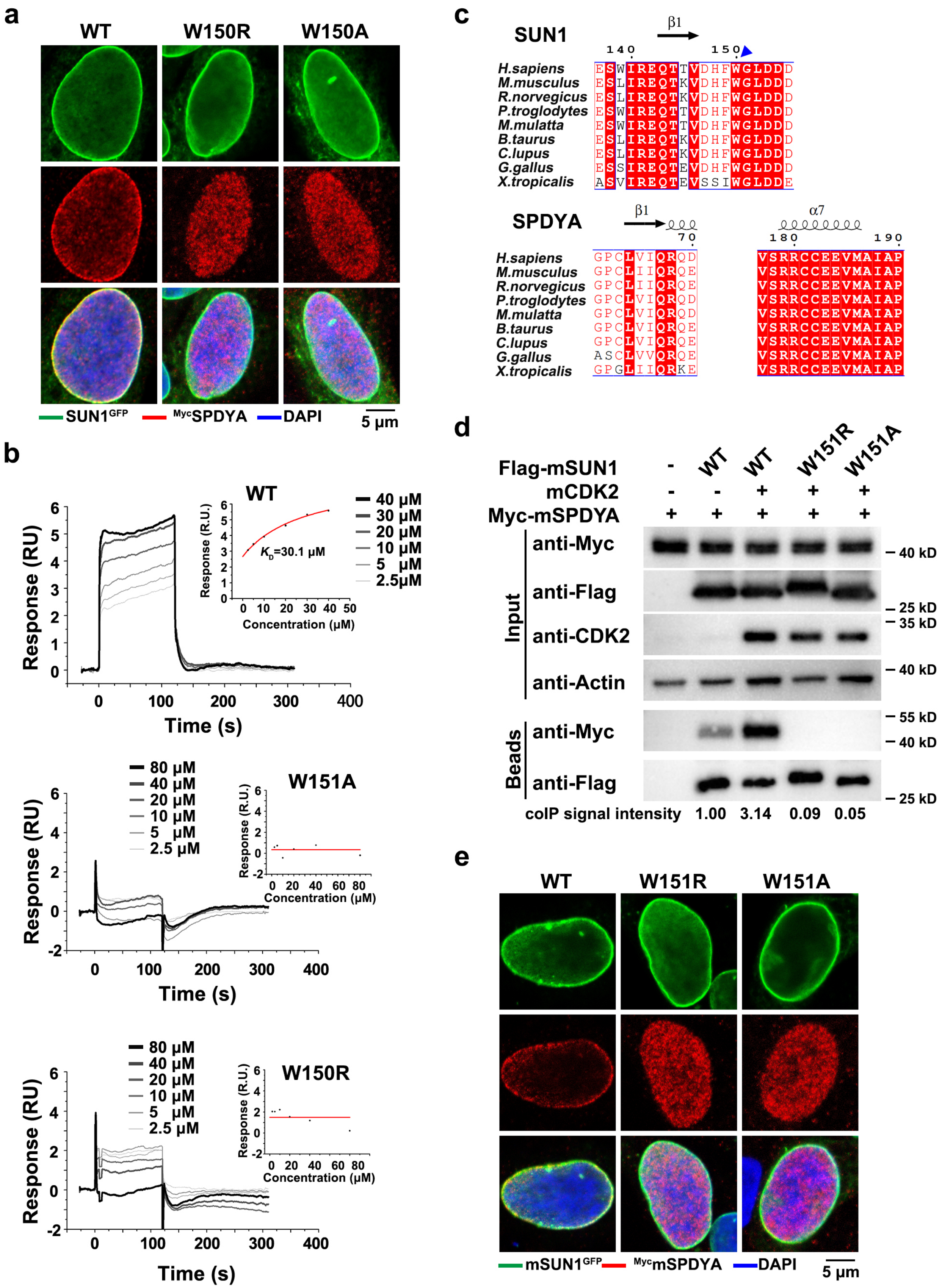

a
WT
W150R
W150A
SUN1GFP
MycSPDYA
DAPI
5 μm
b
WT
K_D=30.1 μM
40 μM
30 μM
20 μM
10 μM
5 μM
2.5μM
Response (RU)
Response (R.U.)
Concentration (μM)
Time (s)
W151A
80 μM
40 μM
20 μM
10 μM
5 μM
2.5 μM
W150R
c
SUN1
β1
140
150
H.sapiens
M.musculus
R.norvegicus
P.troglodytes
M.mulatta
B.taurus
C.lupus
G.gallus
X.tropicalis
SPDYA
α7
70
180
190
d
Flag-mSUN1
mCDK2
Myc-mSPDYA
WT
W151R
W151A
Input
Beads
anti-Myc
anti-Flag
anti-CDK2
anti-Actin
40 kD
25 kD
35 kD
55 kD
coIP signal intensity 1.00 3.14 0.09 0.05
e
WT
W151R
W151A
mSUN1GFP
MycmSPDYA
DAPI
5 μm

Supplementary Figure 3: Mutational analysis of the SUN1-SPDYA interface.

a, Equator images of U-2 OS cells expressing human SUN1-GFP (green) and Myc-SPDYA. Cells were immunostained with anti-Myc antibody (red). Scale bar, 5 μm. **b**, Surface plasmon resonance measurements showing that WT $SUN1_{131\text{-}160}$ fragment bound to the $SPDYA_{ERD}$-CDK2 complex in a concentration-dependent manner. Graphs of equilibrium response unit versus SUN1 concentrations were plotted. The estimated K_D for WT interaction is 30.1 μM. **c**, Multiple sequence alignments of SUN1 (residues 137-155) and SPDYA (residues 61-70 and 177-190) homologs from various species. Sequence positions are shown based on the human SUN1 and human SPDYA, respectively. SUN1 sequences used for alignment are from: *H. sapiens*, O94901; *M. musculus*, Q9D666; *R. norvegicus*, Q5U2W0; *P. troglodytes*, H2QU30; *M. mulatta*, F7HLL7; *B. taurus*, G3MWU2; *C. lupus*, E2R8J5; *G. gallus*, E1BQ86; *X. tropicalis*, A0A5G3KJ67. SPDYA sequences are from: *H. sapiens*, Q5MJ70; *M. musculus*, Q5IBH7; *R. norvegicus*, Q8R496; *P. troglodytes*, A0A2J8K0K9; *M. mulatta*, F6Y2M5; *B. taurus*, F1MBL9; *C. lupus*, J9P8P8; *G. gallus*, E1C1Y3; *X. tropicalis*, A8E5U5. **d**, Co-IP of ectopically expressed mouse Flag-$SUN1_{1\text{-}210}$, Myc-SPDYA and CDK2 in HEK293T cells. The immunoprecipitation was performed with anti-Flag beads. The levels of each protein in the input and IP samples were analyzed by immunoblotting with the indicated antibodies. Band intensity of blots was quantified by the ImageJ software and the co-IP signal for the sample with expression of WT Flag-$SUN1_{1\text{-}210}$ and Myc-SPDYA was set as 1. **e**, Equator images of U-2 OS cells expressing mouse SUN1-GFP (green) and Myc-SPDYA. Cells were immunostained with anti-Myc antibody (red). Scale bar, 5 μm. Source data are provided as a Source Data file.

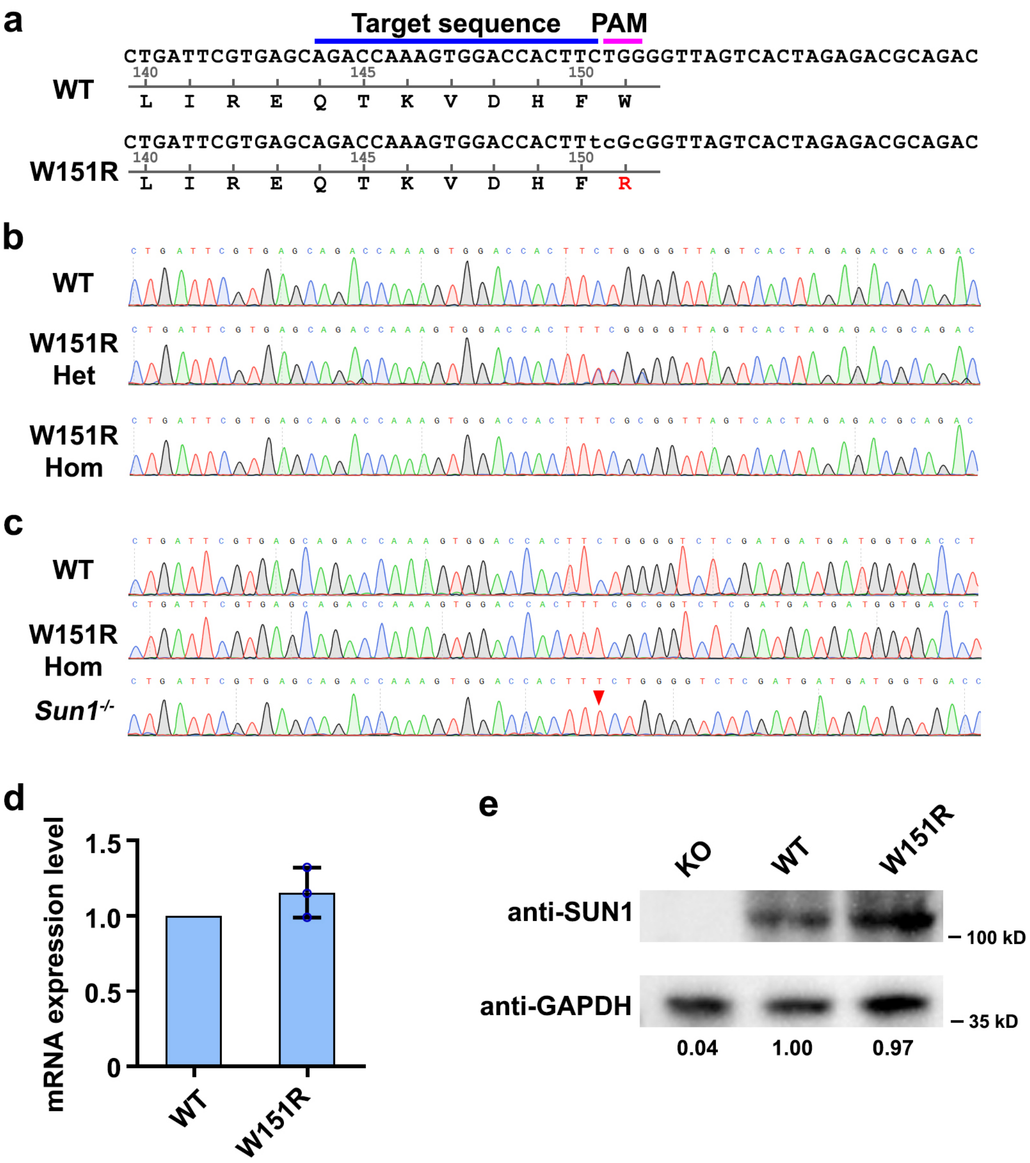

a
Target sequence
PAM
WT
CTGATTCGTGAGCAGACCAAAGTGGACCACTTCTGGGGTTAGTCACTAGAGACGCAGAC
140
145
150
L I R E Q T K V D H F W
W151R
CTGATTCGTGAGCAGACCAAAGTGGACCACTTtcGcGGTTAGTCACTAGAGACGCAGAC
140
145
150
L I R E Q T K V D H F R
b
WT
W151R Het
W151R Hom
c
WT
W151R Hom
Sun1-/-
d
mRNA expression level
1.5
1.0
0.5
0
WT
W151R
e
KO
WT
W151R
anti-SUN1
100 kD
anti-GAPDH
35 kD
0.04
1.00
0.97

Supplementary Figure 4: Targeted disruption of SUN1-SPDYA interaction in mice.

a, The Cas9/sgRNA-targeting site for mouse *Sun1* mutation. The sgRNA-targeting site and the protospacer adjacent motif (PAM) are indicated above the sequence. The desired mutation on genomic DNA is indicated by lower-case letters. The altered amino acid residue is indicated in red color. **b**, Mouse offspring were genotyped by tail biopsy with PCR and DNA sequencing of the mutation loci. Het, heterozygote. Hom, homozygote. **c**, Testis cDNA library from homozygous mice was subjected to PCR and DNA sequencing to confirm the desired mutations. $Sun1^{-/-}$ mice contain a frameshift insertion at His149 (arrowhead). **d**, Relative mRNA levels of *Sun1* were determined by qPCR with testes from 2-week-old WT and mutant homozygous mice. Data are presented as the mean ± SD (n = 3 independent experiments with different mouse littermates). For each experiment, the relative mRNA levels of *Sun1* for WT was set as 1. **e**, Western blotting of SUN1 in testes of 2-week-old mice. GADPH was used as the loading control. Band intensity of blots was quantified by the ImageJ software and the signal for the WT sample was set as 1. Source data are provided as a Source Data file.

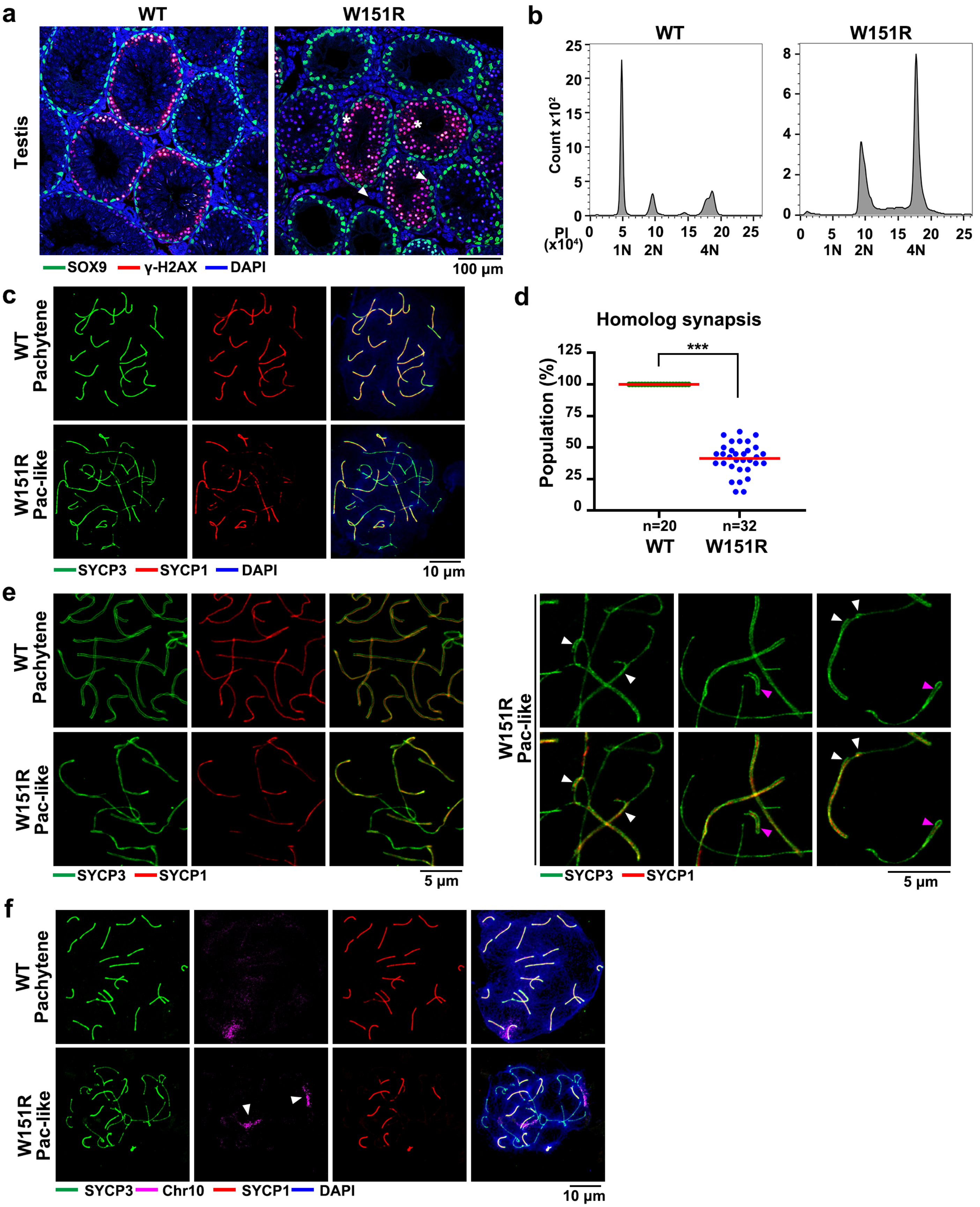
a
WT
W151R
Testis
SOX9
γ-H2AX
DAPI
100 µm
b
WT
W151R
Count x10²
PI (x10⁴)
1N
2N
4N
c
WT Pachytene
W151R Pac-like
SYCP3
SYCP1
DAPI
10 µm
d
Homolog synapsis
Population (%)
***
n=20
n=32
WT
W151R
e
WT Pachytene
W151R Pac-like
SYCP3
SYCP1
5 µm
W151R Pac-like
SYCP3
SYCP1
5 µm
f
WT Pachytene
W151R Pac-like
SYCP3
Chr10
SYCP1
DAPI
10 µm

Supplementary Figure 5: The SUN1-SPDYA interaction is indispensable for prophase I progression.

a, IF staining for SOX9 (green) and γ-H2AX (red) of testis sections from adult mice. Arrowheads indicate abnormal spermatocyte-like cells with persistent γ-H2AX signal in mutant seminiferous tubules. Asterisks show seminiferous containing a single layer of sertoli cells and very few spermatogonial cells at the tubule periphery. DNA was stained by DAPI (blue). Scale bar, 100 μm. **b**, Propidium iodide (PI) fluorescence flow analysis of fixed testicular cells from 6-week-old male mice. Cells were stained by PI. 1N, haploid; 2N, diploid; 4N, tetraploid. **c**, IF staining of fetal oocyte chromosome spreads for SYCP3 (green) and SYCP1 (red). (Pac-like: Pachytene-like). Scale bar, 10 μm. **d**, Population of fully paired homologous chromosomes in each WT pachytene or *W151R* pachytene-like ovaries from panel **c**. A total of n=20 WT and n=32 *W151R* ovary spreads were counted. Red lines indicated the mean values. A two-sided Student's *t*-test was performed, ** $p = 7.9E\text{-}27$. **e**, IF analysis by STED of SYCP3 (green) and SYCP1 (red) on spermatocyte spreads from adult mouse testes. Below: Representative STED images of *W151R* spreads showing aberrant synapsis. White arrowheads indicate pairing between non-homologous chromosomes and the purple arrowheads show self-pairing. Scale bars, 5 μm. **f**, IF staining of SYCP3 (green) and SYCP1 (red) and FISH detection of chromosome 10 on spermatocyte spreads from adult testes of different genotypes. Scale bar, 10 μm. Source data are provided as a Source Data file.

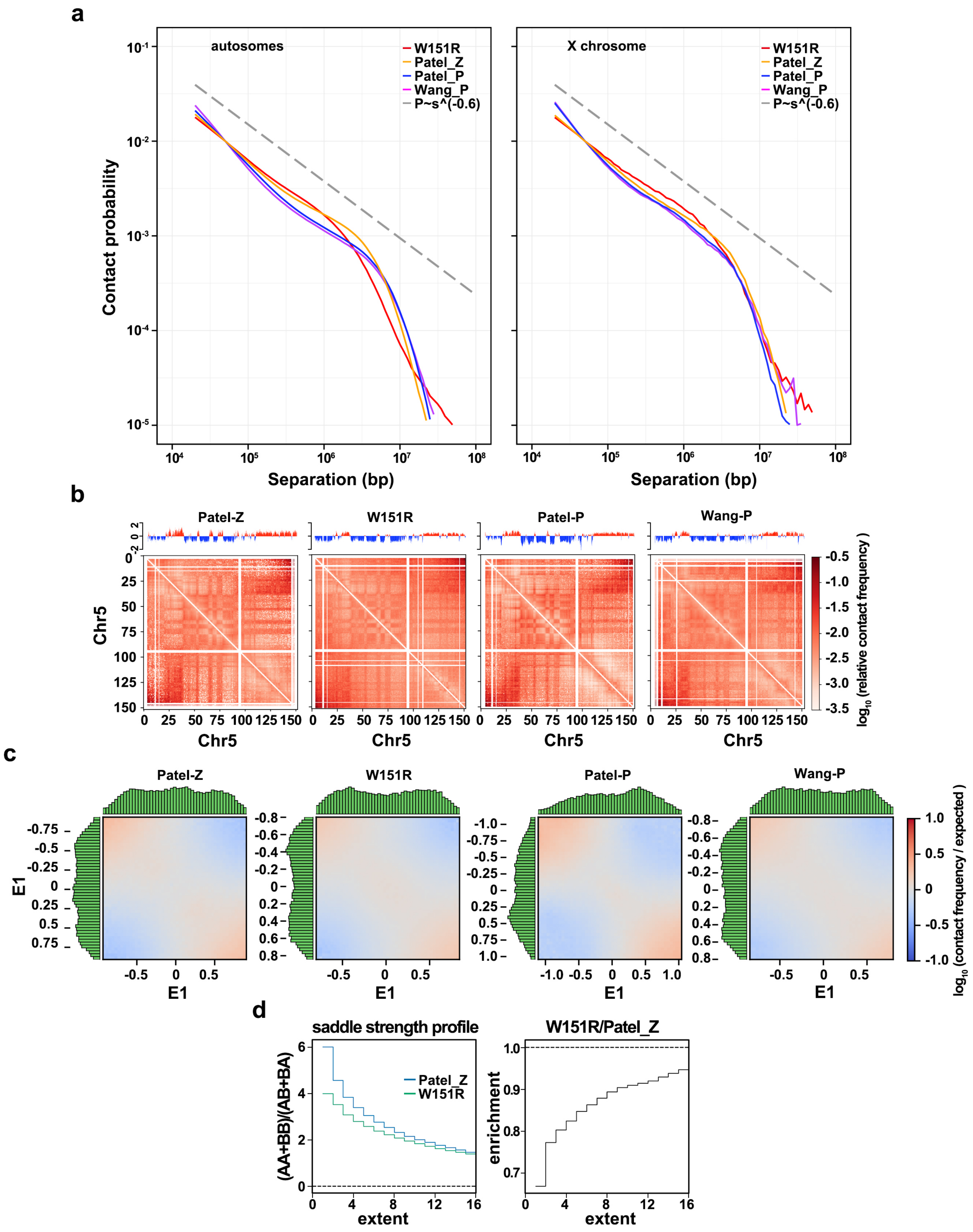

a
autosomes
X chrosome
W151R
Patel_Z
Patel_P
Wang_P
P~s^(-0.6)
Contact probability
Separation (bp)
b
Patel-Z
W151R
Patel-P
Wang-P
Chr5
log10 (relative contact frequency)
c
E1
log10 (contact frequency / expected)
d
saddle strength profile
(AA+BB)/(AB+BA)
extent
W151R/Patel_Z
enrichment

Supplementary Figure 6: Disruption of the SUN1-SPDYA interaction affects 3D genome organization.

a, P(s) curves showing relationships between chromatin contact probability and genomic separation for chromatin interactions on autosomes (left) and the X chromosome (right) in WT zygotene and pachytene spermatocytes as well as *W151R* mutant spermatocytes. Dotted lines corresponding to $P(s) \sim s^{-0.6}$ and $P(s) \sim s^{-1.2}$ are shown as references. WT data included in these analyses are from Patel et al (Patel_Z and Patel_P)[25] and Wang et al (Wang_P)[26]. **b**, Heatmaps binned at 50 kb resolution showing normalized Hi-C interactions (observed/expected) for Chr5. Plots of eigenvector 1 values on top of heatmaps indicate positions of A (red) and B (blue) compartments. The A/B compartment identities for genomic regions are largely unchanged across different samples. **c**, Saddle plots indicating the extent of genome compartmentalization in each dataset. **d**, Saddle strength profiles quantifying the strength of genome compartmentalization by calculating ratios of cumulative corner interaction scores from the saddle plots ((AA+BB)/(AB+BA)). Compared to WT zygotene spermatocytes, the *W151R* mutant spermatocytes exhibit weaker compartmentalization.

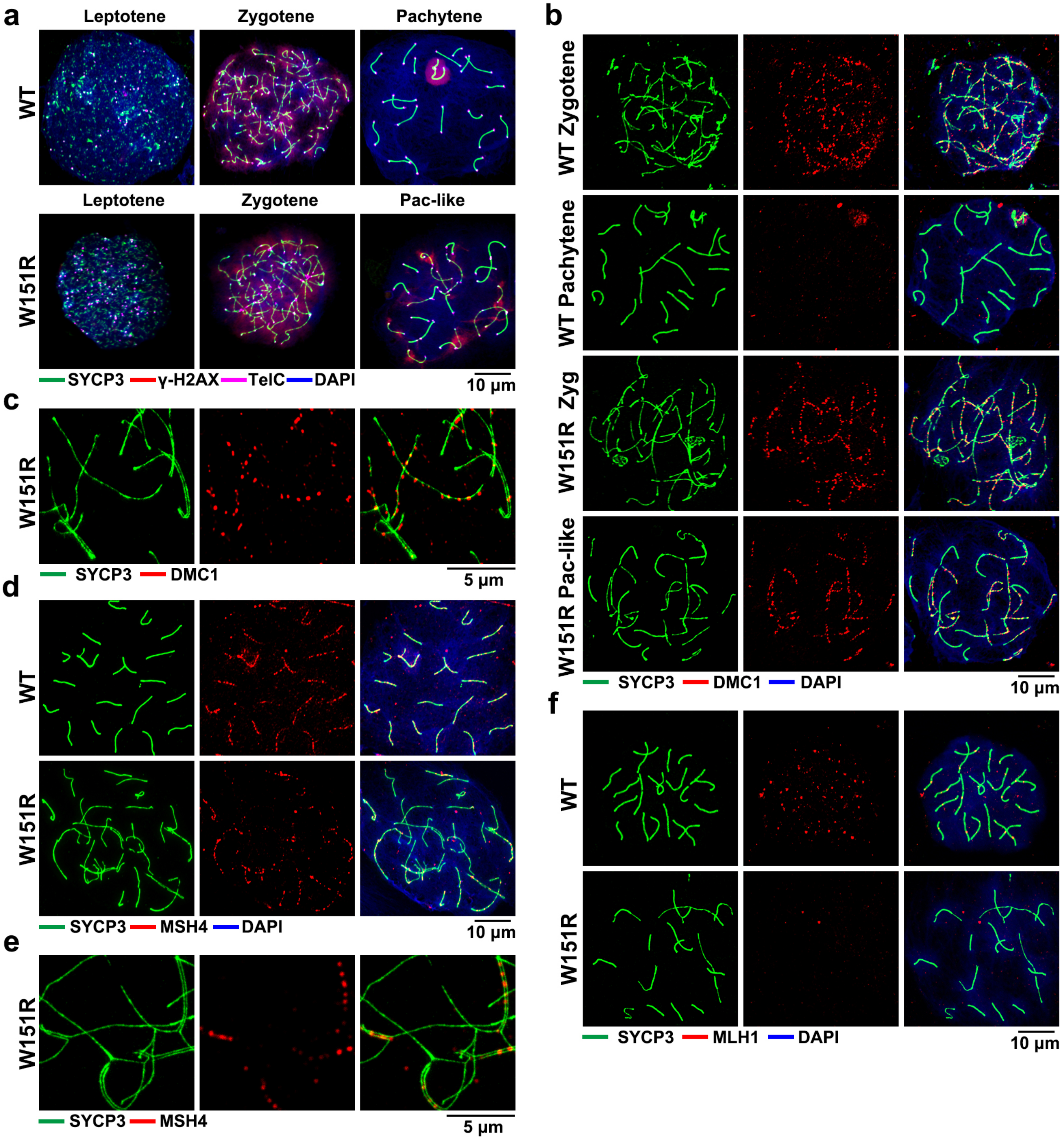
a
Leptotene
Zygotene
Pachytene
WT
Leptotene
Zygotene
Pac-like
W151R
SYCP3
γ-H2AX
TelC
DAPI
10 μm
b
WT Zygotene
WT Pachytene
W151R Zyg
W151R Pac-like
SYCP3
DMC1
DAPI
10 μm
c
W151R
SYCP3
DMC1
5 μm
d
WT
W151R
SYCP3
MSH4
DAPI
10 μm
e
W151R
SYCP3
MSH4
5 μm
f
WT
W151R
SYCP3
MLH1
DAPI
10 μm

Supplementary Figure 7: Disruption of the SUN1-SPDYA interaction interferes with meiotic DSB repair.

a, Representative spermatocyte nuclei from 6-week-old mice at different indicated stages stained with anti-SYCP3 antibody (green) and anti-γ-H2AX antibody (red). DNA was stained by DAPI (blue). **b**, IF analysis of spermatocyte chromosome spreads stained with anti-SYCP3 antibody (green) and anti-DMC1 antibody (red). **c**, IF analysis by STED of *W151R* spermatocyte spreads stained with anti-SYCP3 antibody (green) and anti-DMC1 antibody (red). **d**, IF analysis of spermatocyte chromosome spreads stained with anti-SYCP3 antibody (green) and anti-MSH4 antibody (red). **e**, IF analysis by STED of *W151R* spermatocyte spreads stained with anti-SYCP3 antibody (green) and anti-MSH4 antibody (red). **f**, IF analysis of spermatocyte chromosome spreads stained with anti-SYCP3 antibody (green) and anti-MLH1 antibody (red).

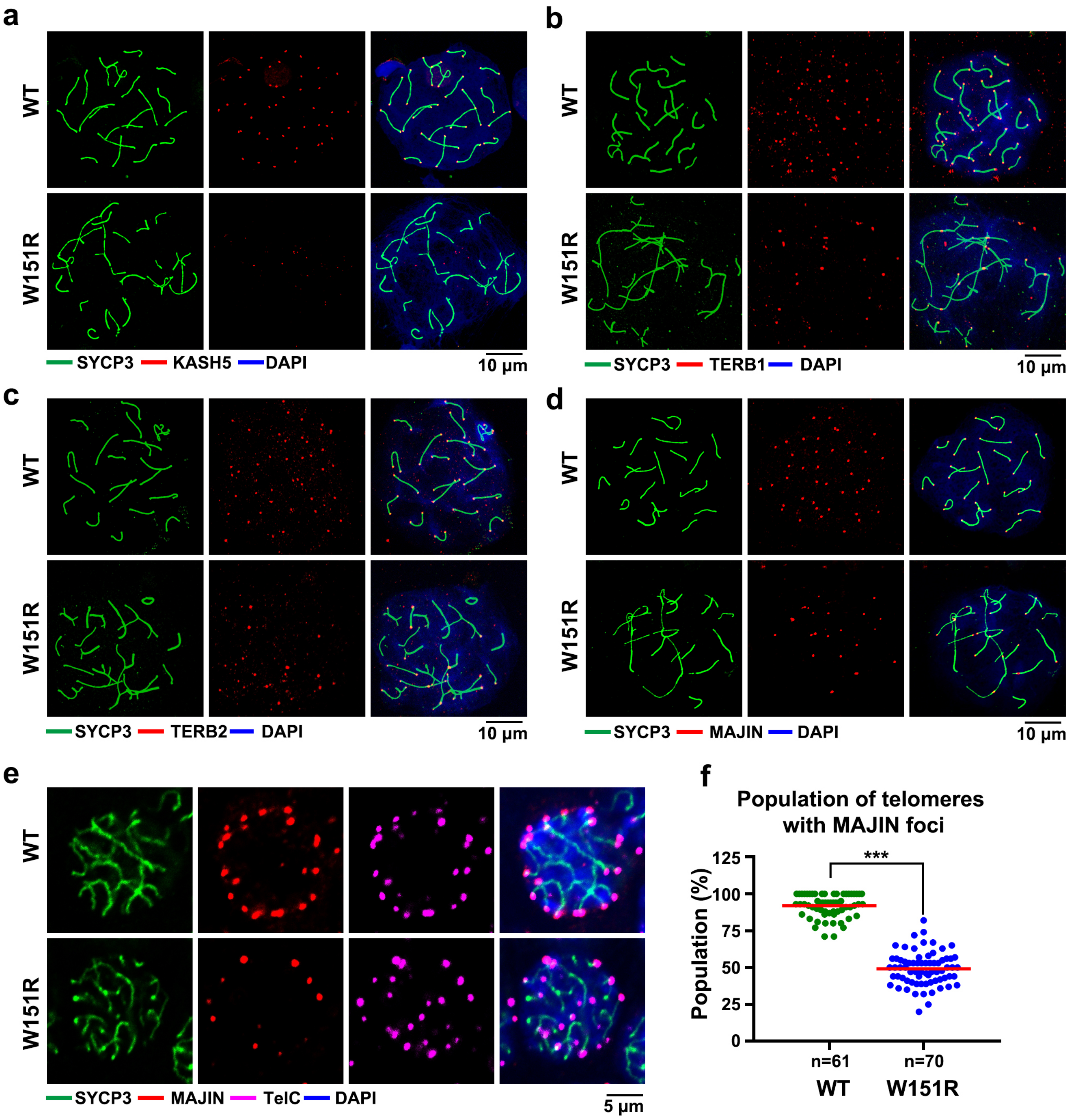
a
WT
W151R
SYCP3 KASH5 DAPI
10 μm
b
WT
W151R
SYCP3 TERB1 DAPI
10 μm
c
WT
W151R
SYCP3 TERB2 DAPI
10 μm
d
WT
W151R
SYCP3 MAJIN DAPI
10 μm
e
WT
W151R
SYCP3 MAJIN TelC DAPI
5 μm
f
Population of telomeres with MAJIN foci
Population (%)
125
100
75
50
25
0
***
n=61
n=70
WT
W151R

Supplementary Figure 8: Telomeres are partially retained at the nuclear periphery with TTM in mutant spermatocytes.

a-**d**, IF staining of spermatocyte chromosome spreads for SYCP3 (green) and KASH5 (red), TERB1 (red), TERB2 (red), or MAJIN (red) as indicated. DNA was stained by DAPI (blue). **e**, Equator images of structurally preserved zygotene spermatocytes stained for SYCP3 (green), MAJIN (red) and telomere FISH (TelC, magenta). Scale bar, 5 μm. **f**, Population of telomeres displaying MAJIN foci in each spermatocyte nucleus as shown in panel **e**. A total of n=61 WT and n=70 *W151R* spermatocytes were counted. Red lines indicated the mean values. A two-sided Student's *t*-test was performed, *** *p* =4.7E-51. Source data are provided as a Source Data file.

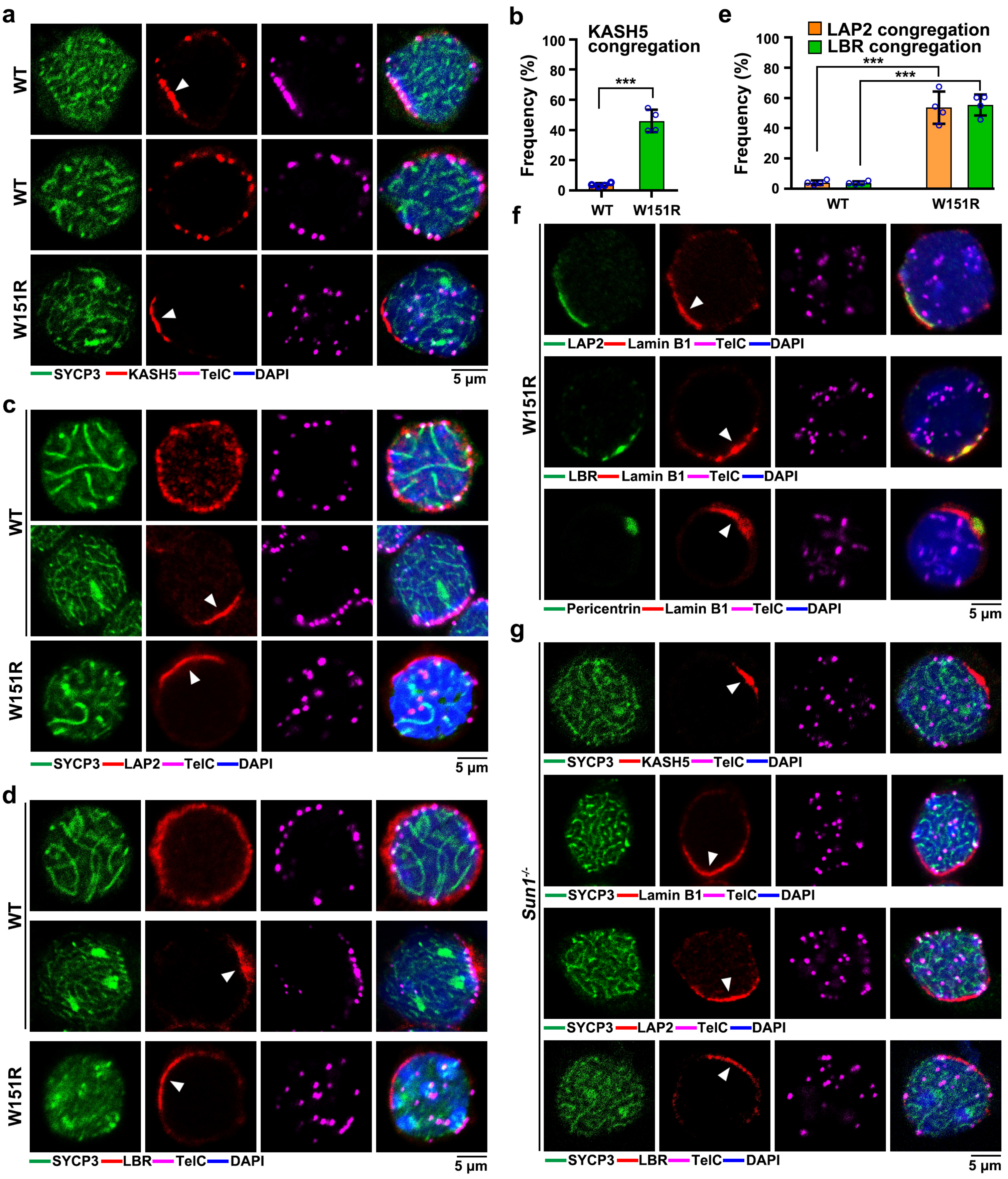

a
WT
WT
W151R
SYCP3 KASH5 TelC DAPI
5 μm
b
KASH5 congregation
Frequency (%)
100
80
60
40
20
0
***
WT
W151R
e
LAP2 congregation
LBR congregation
Frequency (%)
100
80
60
40
20
0
***
***
WT
W151R
c
WT
W151R
SYCP3 LAP2 TelC DAPI
5 μm
d
WT
W151R
SYCP3 LBR TelC DAPI
5 μm
f
W151R
LAP2 Lamin B1 TelC DAPI
LBR Lamin B1 TelC DAPI
Pericentrin Lamin B1 TelC DAPI
5 μm
g
Sun1-/-
SYCP3 KASH5 TelC DAPI
SYCP3 Lamin B1 TelC DAPI
SYCP3 LAP2 TelC DAPI
SYCP3 LBR TelC DAPI
5 μm

Supplementary Figure 9: Disruption of the SUN1-SPDYA interaction interferes with the distribution of the LINC complex and lamina at the NE.

a, Equator images of structurally preserved zygotene spermatocytes stained for SYCP3 (green), KASH5 (red) and telomeric DNA (TelC probe, magenta). Arrowheads indicate clustered signals for KASH5. DNA was stained with DAPI (blue). Scale bar, 5 µm. **b**, Population of nuclei displaying KASH5 congregation in panel **a**. Data are presented as the mean ± SD (n = 4 mice of each indicated genotype). A two-sided Student's *t*-test was performed, *** $p = 2.9E\text{-}5$. More than 100 spermatocytes from each indicated genotype were counted. **c**, Equator images of structurally preserved zygotene spermatocytes stained for SYCP3 (green), LAP2 (red) and telomeric DNA (TelC probe, magenta). Arrowheads indicate congregated signals for LAP2. Scale bar, 5 µm. **d**, Equator images of structurally preserved zygotene spermatocytes stained for SYCP3 (green), LBR (red) and telomeric DNA (TelC probe, magenta). Arrowheads indicate congregated signals for LBR. Scale bar, 5 µm. **e**, Population of nuclei exhibiting LAP2 or LBR congregation in panels **c** and **d**, respectively. Data are presented as the mean ± SD (n = 4 mice of each indicated genotype). A two-sided Student's *t*-test was performed, *** $p = 9.2E\text{-}5$ (LAP2) and *** $p = 6.2E\text{-}6$ (LBR). More than 100 spermatocytes from each indicated genotype were counted. **f**, Equator images of structurally preserved zygotene spermatocytes stained with indicated antibodies and telomere FISH probe. Arrowheads indicate congregated signals for Lamin B1. Scale bar, 5 µm. **g**, Equator images of structurally preserved zygotene spermatocytes stained with indicated antibodies and telomere FISH probe. Arrowheads indicate congregated signals for KASH5, Lamin B1, LAP2 and LBR, respectively. Scale bar, 5 µm. Source data are provided as a Source Data file.

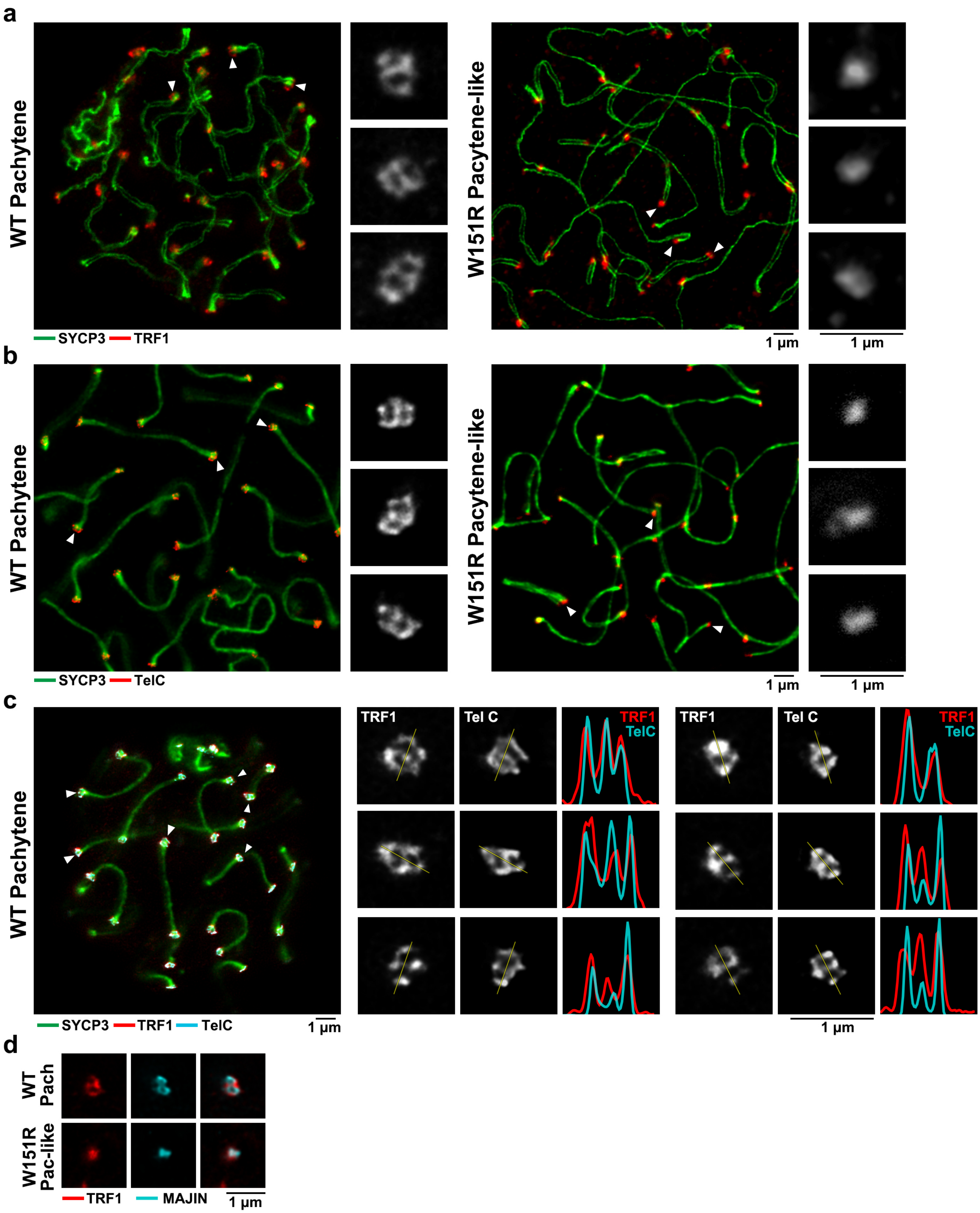
a
WT Pachytene
SYCP3
TRF1
W151R Pacytene-like
1 µm
1 µm
b
WT Pachytene
SYCP3
TelC
W151R Pacytene-like
1 µm
1 µm
c
WT Pachytene
SYCP3
TRF1
TelC
1 µm
TRF1
Tel C
TRF1
TelC
TRF1
Tel C
TRF1
TelC
1 µm
d
WT Pach
W151R Pac-like
TRF1
MAJIN
1 µm

Supplementary Figure 10: Meiotic telomere structures at the INM.

a, IF analysis by STED of spermatocyte chromosome spreads stained for SYCP3 (green) and TRF1 (red). Insets are enlarged figures of telomere architectures indicated by arrowheads. Although *W151R* pachytene-like spermatocytes exhibited both asynapsed axial elements (AEs) and synapsed lateral elements (LEs), no ring-shaped structures were observed at chromosome tips. Scale bar: 1 μm. **b**, IF-FISH analysis of spermatocyte chromosome spreads stained for SYCP3 (green) and telomeric DNA (telC, red). Insets are enlarged figures of telomere architectures indicated by arrowheads. No ring-shaped structures were observed at chromosome tips. Scale bar: 1 μm. **c**, Co-localization of TRF1 and telomeric DNA revealed by STED microscopic analysis. Scale bar: 1 μm. **d**, IF analysis by STED of spermatocyte spreads stained for TRF1 (red) and MAJIN (cyan). Scale bar, 1 μm.

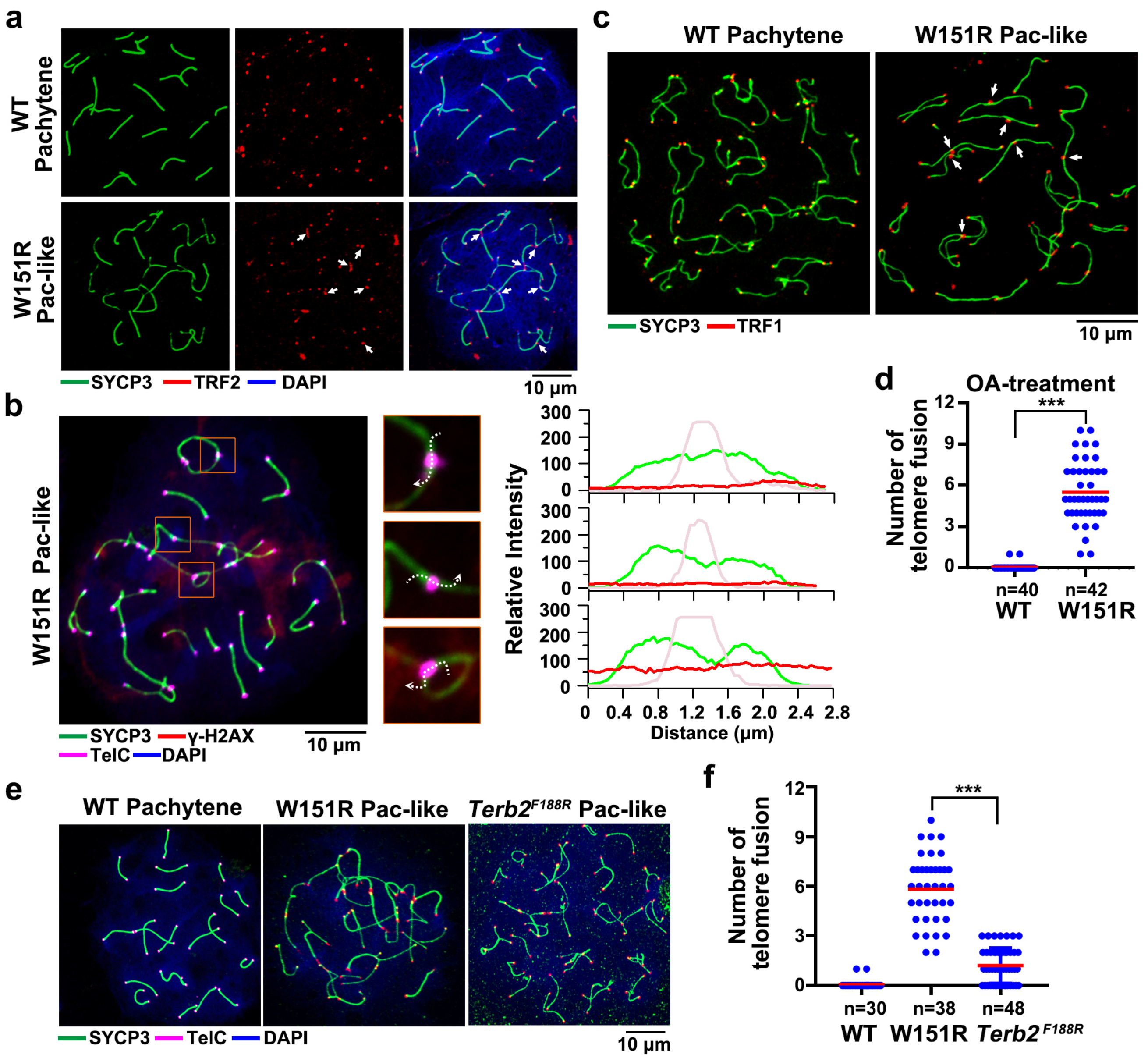

a
WT Pachytene
W151R Pac-like
SYCP3 TRF2 DAPI
10 μm
b
W151R Pac-like
SYCP3 γ-H2AX
TelC DAPI
10 μm
Relative Intensity
Distance (μm)
c
WT Pachytene
W151R Pac-like
SYCP3 TRF1
10 μm
d
OA-treatment
***
Number of telomere fusion
n=40
WT
n=42
W151R
e
WT Pachytene
W151R Pac-like
Terb2F188R Pac-like
SYCP3 TelC DAPI
10 μm
f
***
Number of telomere fusion
n=30
WT
n=38
W151R
n=48
Terb2F188R

Supplementary Figure 11: Disruption of the SUN1-SPDYA interaction causes telomere fusions.

a, IF analysis of spermatocyte spreads from adult mice stained for SYCP3 (green) and TRF2 (red). DNA was stained by DAPI (blue). Arrowheads indicate telomere fusions and bridges. Scale bar, 10 μm. **b**, IF-FISH analysis of mutant spermatocyte spreads stained for SYCP3 (green), γ-H2AX (red) and telomeric DNA (telC, magenta). Telomere fusions shown in the framed regions were analyzed for the signal distribution of SYCP3, γ-H2AX and telomeric DNA. Scale bar, 10 μm. **c**, IF analysis of spermatocyte spreads from adult mice stained with indicated antibodies. Spermatocytes were incubated with 5 μM Okadaic acid (OA) at 30 °C for 2 hours prior to chromosome spread preparation. Arrowheads indicate telomere fusions and bridges. Scale bar, 10 μm. **d**, Quantification of number of telomere fusions and bridges per nucleus of WT (n = 40) and *W151R* (n = 42) spermatocytes in **c**. Red lines indicated the mean values. A two-sided Student's *t*-test was performed, *** $p = 1.9E\text{-}25$. **e**, IF-FISH images of chromosome spreads stained with indicated antibodies and telomere FISH probe. Scale bar, 10 μm. **f**, Quantification of number of telomere fusions and bridges per nucleus of WT (n = 30), *W151R* (n = 38) and *Terb2*^{F188R} spermatocytes in panel **e**. Red lines indicated the mean values. A two-sided Student's *t*-test was performed, *** $p = 6.3E\text{-}23$. Source data are provided as a Source Data file.

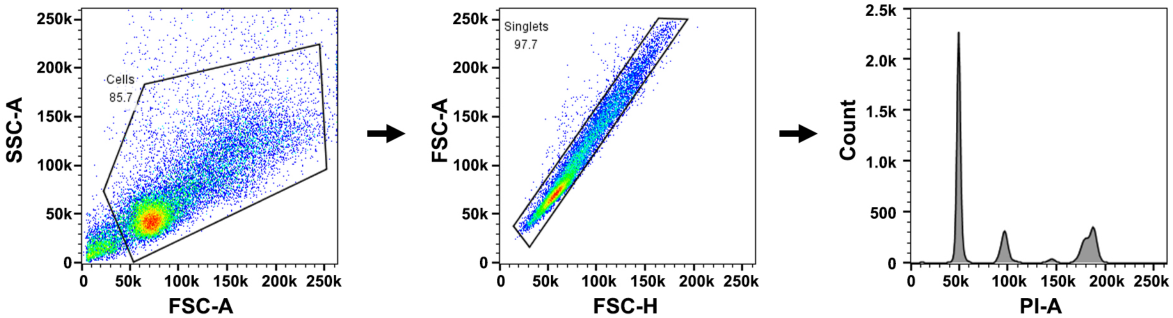

Cells
85.7
SSC-A
FSC-A
Singlets
97.7
FSC-A
FSC-H
Count
PI-A

Supplementary Figure 12: FACS gating strategy example for Supplementary Fig. 5b

Testicular cells were first gated by FSC-A vs SSC-A to eliminate any debris, then gated for singlets by FSC-H vs FSC-A. spermatocytes were then determined from this gated population by PI staining.
