# Supplementary material for: The SUN1-SPDYA interaction plays an essential role in meiosis prophase I
Source: Nat Commun. 2021 May 26;12:3176. doi: 10.1038/s41467-021-23550-w (PMC8155084; doi:10.1038/s41467-021-23550-w)
Supplement: Supplementary file 3 — Description of Additional Supplementary Files [file 41467_2021_23550_MOESM3_ESM.pdf]

Description of Additional Supplementary Files

File Name: Supplementary Data 1
Description: List of primers.
