# Supplementary material for: The SUN1-SPDYA interaction plays an essential role in meiosis prophase I
Source: Nat Commun. 2021 May 26;12:3176. doi: 10.1038/s41467-021-23550-w (PMC8155084; doi:10.1038/s41467-021-23550-w)
Supplement: Supplementary file 5 — Reporting Summary [file 41467_2021_23550_MOESM5_ESM.pdf]

nature research

Corresponding author(s): Ming Lei, Chenhui Huang

Last updated by author(s): Apr 16, 2021

Reporting Summary

Nature Research wishes to improve the reproducibility of the work that we publish. This form provides structure for consistency and transparency in reporting. For further information on Nature Research policies, see our Editorial Policies and the Editorial Policy Checklist.

Statistics

For all statistical analyses, confirm that the following items are present in the figure legend, table legend, main text, or Methods section.

n/a	Confirmed	
☐	☒	The exact sample size (*n*) for each experimental group/condition, given as a discrete number and unit of measurement
☐	☒	A statement on whether measurements were taken from distinct samples or whether the same sample was measured repeatedly
☐	☒	The statistical test(s) used AND whether they are one- or two-sided<br>*Only common tests should be described solely by name; describe more complex techniques in the Methods section.*
☒	☐	A description of all covariates tested
☒	☐	A description of any assumptions or corrections, such as tests of normality and adjustment for multiple comparisons
☐	☒	A full description of the statistical parameters including central tendency (e.g. means) or other basic estimates (e.g. regression coefficient) AND variation (e.g. standard deviation) or associated estimates of uncertainty (e.g. confidence intervals)
☐	☒	For null hypothesis testing, the test statistic (e.g. F, t, r) with confidence intervals, effect sizes, degrees of freedom and P value noted<br>*Give P values as exact values whenever suitable.*
☒	☐	For Bayesian analysis, information on the choice of priors and Markov chain Monte Carlo settings
☒	☐	For hierarchical and complex designs, identification of the appropriate level for tests and full reporting of outcomes
☒	☐	Estimates of effect sizes (e.g. Cohen's d, Pearson's r), indicating how they were calculated

Our web collection on statistics for biologists contains articles on many of the points above.

Software and code

Policy information about availability of computer code

Data collection	no software was used.
Data analysis	Coot version 0.89 (Emsley and Cowtan, 2004)<br>PyMOL version 1.86 (Schrödinger)<br>ImageJ version 1.52 (NIH)<br>FlowJo version 10 (Tree Star)<br>cooler version 0.8.5 (https://github.com/open2c/cooler)<br>cooltools version 0.2.0 (https://github.com/open2c/cooltools)<br>GraphPad Prism v7.04 (GraphPad)<br>Biacore 8K Evaluation Software version 1.0

For manuscripts utilizing custom algorithms or software that are central to the research but not yet described in published literature, software must be made available to editors and reviewers. We strongly encourage code deposition in a community repository (e.g. GitHub). See the Nature Research guidelines for submitting code & software for further information.

Data

Policy information about availability of data

All manuscripts must include a data availability statement. This statement should provide the following information, where applicable:

- Accession codes, unique identifiers, or web links for publicly available datasets
- A list of figures that have associated raw data
- A description of any restrictions on data availability

The crystal structure of the SUN1SBM-SPDYAERD-CDK2 complex was deposited in PDB with accession code 7E34 [https://doi.org/10.2210/pdb7E34/pdb]. The Hi-C

data (Sun1 W151R pachytene-like spermatocytes) was deposited in the Gene Expression Omnibus database with accession code GSE155142 [https://www.ncbi.nlm.nih.gov/geo/query/acc.cgi?acc=GSE155142]. Other data supporting the findings of this study are available from the corresponding author on reasonable request. Source data are provided with this paper.

Field-specific reporting

Please select the one below that is the best fit for your research. If you are not sure, read the appropriate sections before making your selection.

[x] Life sciences [ ] Behavioural & social sciences [ ] Ecological, evolutionary & environmental sciences

For a reference copy of the document with all sections, see nature.com/documents/nr-reporting-summary-flat.pdf

Life sciences study design

All studies must disclose on these points even when the disclosure is negative.

Sample size	For animal experiments, no statistical method was used to predetermine sample size. For in vivo analysis, a minimum of n=3 mice of each genotype were used and several cells (nuclei, spreads) or hundreds of telomeres from each mouse were analyzed. This was shown to be sufficient in previous studies (e.g.,Shibuya et al Cell 2015, Mikolcevic et al Nat Commun 2016) to discern statistically significant differences. In molecular biology experiments, n>=3 was chosen to generate p-values to determine if results are significant.
Data exclusions	No data were excluded.
Replication	The in vitro data were reproduced in technical and biological duplicates and experiments were performed at least three times. The exact number of mice used in each group in an experiment is mentioned in corresponding figure legends. All attempts at replication were successful.
Randomization	For mouse analysis, we are comparing phenotypes between WT and SUN1W151R pups. Cells and sections used for imaging were selected randomly. For in vitro experiments, Cells for imaging were selected randomly.
Blinding	Blinding was not possible as experimental set-up and data-analysis were performed by the same individuals.

Reporting for specific materials, systems and methods

We require information from authors about some types of materials, experimental systems and methods used in many studies. Here, indicate whether each material, system or method listed is relevant to your study. If you are not sure if a list item applies to your research, read the appropriate section before selecting a response.

Materials & experimental systems

n/a	Involved in the study	
[ ]	[x]	Antibodies
[ ]	[x]	Eukaryotic cell lines
[x]	[ ]	Palaeontology and archaeology
[ ]	[x]	Animals and other organisms
[x]	[ ]	Human research participants
[x]	[ ]	Clinical data
[x]	[ ]	Dual use research of concern

Methods

n/a	Involved in the study	
[x]	[ ]	ChIP-seq
[ ]	[x]	Flow cytometry
[x]	[ ]	MRI-based neuroimaging

Antibodies

Antibodies used	The following antibodies were used in this study: secondary antibodies for IF and western blotting: Alexa Fluor 647 Goat anti-Rabbit IgG (Invitrogen, A-21245, 1:500, RRID: AB_2535813), Alexa Fluor 647 Goat anti-Mouse IgG (Invitrogen, A-21236, 1:500, RRID: AB_2535805), Alexa Fluor 555 Goat anti-Rabbit IgG (Invitrogen, A-21429, 1:500, RRID: AB_2535850), Alexa Fluor 555 Goat anti-Mouse IgG (Invitrogen, A-21424, 1:500, RRID: AB_141780), Alexa Fluor 488 Goat anti-Rabbit IgG (Invitrogen, A-11008, 1:500, RRID: AB_143165), Alexa Fluor 488 Goat anti-Mouse IgG (Invitrogen, A-11001, 1:500, RRID: AB_2534069), DyLight 594 Goat anti-Mouse IgG (Invitrogen, 35511, 1:500, RRID: AB_1965950), DyLight 594 Goat anti-Rabbit IgG (Invitrogen, 35561, 1:500, RRID: AB_1965951), HRP-conjugated Goat anti-Mouse IgG (Proteintech, SA00001-1, 1:4000, RRID: AB_2722565), and HRP-conjugated Goat anti-Rabbit IgG (Proteintech, SA00001-2, 1:4000, RRID: AB_2722564). Rabbit antibodies against SYCP1 (Abcam, ab15087, 1:2000, RRID: AB_301633), SYCP3 (Abcam, ab15093, 1:2000, RRID: AB_301639), SOX9 (Millipore, ab5535, 1:500, RRID: AB_2239761), MSH4 (Abcam, ab58556, 1:500, RRID: AB_2770446), phos-Histone H2AX (Ser139) (Millipore, 05-636, 1:1000, RRID: AB_309864), Lamin B1 (Abcam, ab16048, 1:1000, RRID: AB_443298), c-Myc (Santa Cruz, sc-789, 1:500, RRID: AB_631274), TERB1(Long et al NSMB 2017, 1:1000), TERB2 (Wang et al NC 2019, 1:2000), MAJIN (Wang et al NC 2019, 1:2000), SUN1 (Wang et al NC 2019, 1:1000), and SPDYA (Wang et al NC 2019, 1:3000). Mouse antibodies against Phospho-Thr-Pro (CST, 9391, 1:2000, RRID: AB_331801), MLH1 (BD, 550838, 1:500, RRID: AB_2636290), SYCP3 (Abcam, ab97672, 1:2000, RRID: AB_10678841), c-Myc (Santa cruz, sc-40, 1:500, RRID: AB_627268), Flag (Sigma, F3165, 1:2000, RRID: AB_259529), actin (Sigma, A2228, 1:2000, RRID: AB_476697), GAPDH (Proteintech, 60004-1, 1:2000, RRID: ab_2107436), DMC1 (Abcam, ab11054, 1:500, RRID: AB_297706), Lamin B1 (Proteintech, 66095-1, 1:1000, RRID:

AB_11232208), CDK2 (Santa cruz, sc-6248, 1:200, RRID: AB_627238), Pericentrin (Abcam, ab4448, 1:100, RRID: AB_304461), LBR (Abcam, ab122919, 1:500, RRID: AB_10902156), LAP2 (Abcam, ab185718, 1:500), KASH5 (this study, 1:2000), TRF1 (this study, 1:3000) and TRF2 (this study, 1:3000).

Validation

SYCP1 (Abcam, ab15087) Species Reactivity: Mouse Rat Human, Applications: IHC IF
SYCP3 (Abcam, ab15093) Species Reactivity: Mouse Rat Human, Applications: IHC IF
SOX9 (Millipore, ab5535) Species Reactivity: Mouse Rat Human, Applications: IHC IF WB
MSH4 (Abcam, ab58666) Species Reactivity: Mouse, Applications: ICC IF WB
phos-HistoneH2A.X (Ser139) (Millipore, 05-636-25UG) Species Reactivity: Vertebrates, Applications: IHC IF WB
Lamin B1 (Abcam, ab16048) Species Reactivity: Mouse Rat Human, Applications: IHC IF WB
c-Myc (Santa Cruz, sc-789) Species Reactivity: Mouse Rat Human, c-Myc tagged fusion proteins, Applications: WB, IP, IF
MLH1 (BD, 550838) Species Reactivity: Mouse Rat Human, Applications: IHC IF
SYCP3 (Abcam, ab97672) Species Reactivity: Mouse Hamster, Applications: IHC IF WB
c-myc (Santa cruz, sc-40) Species Reactivity: c-Myc tagged fusion proteins, Applications: WB, IP, IF
FLAG (Sigma, F3165) Species Reactivity: Mouse Rat Human, FLAG tagged fusion proteins, Applications: WB, IP, IF
actin (Sigma, A2228) Species Reactivity: Mouse Rat Human, Applications: WB, IHC
DMC1 (Abcam, ab11054) Species Reactivity: Mouse Rat Human, Applications: IHC IF WB
Lamin B1 (Proteintech, 66095-1-Ig) Species Reactivity: Mouse Rat Human, Applications: IHC IF WB
CDK2 (Santa curz, sc-6248) Species Reactivity: Mouse Rat Human, Applications: IF IP WB
Pericentrin (Abcam, ab4448) Species Reactivity: Mouse Rat Rabbit Human, Applications: IHC IF
LBR (Abcam, ab122919) Species Reactivity: Mouse Human, Applications: WB, IP, IF
LAP2(Abcam, ab185718) Species Reactivity: Mouse Rat Human, Applications: IHC, WB, IF
TERB1 (Long et al NSMB 2017) Species Reactivity: Mouse, Applications: IF WB
TERB2 (Wang et al NC 2019) Species Reactivity: Mouse, Applications: IF WB
MAJIN (Wang et al NC 2019) Species Reactivity: Mouse, Applications: IF WB
SPDYA (Wang et al NC 2019) Species Reactivity: Mouse, Applications: IF WB
SUN1 (Wang et al NC 2019) Species Reactivity: Mouse, Applications: IF WB
KASH5 (this study) Species Reactivity: Mouse, Applications: IF WB
TRF1 (this study) Species Reactivity: Mouse, Applications: IF WB
TRF2 (this study) Species Reactivity: Mouse, Applications: IF WB

Eukaryotic cell lines

Policy information about cell lines

Cell line source(s)

HEK293T and U-2 OS cells were purchased from the Type Culture Collection of the Chinese Academy of Sciences, Shanghai, China.

Authentication

Cells were authenticated by the cell bank of type culture collection of Chinese Academy of Sciences using STR DNA profiling.

Mycoplasma contamination

No mycoplasma contamination was detected in HEK293T and U-2 OS cells used in this study.

Commonly misidentified lines
(See ICLAC register)

No commonly misidentified cell lines were used.

Animals and other organisms

Policy information about studies involving animals; ARRIVE guidelines recommended for reporting animal research

Laboratory animals

Male and female C57BL/6 mice between 6-12 and ICR(CD-1) mice between 8-12 weeks were used in this study. Fertilized eggs collected from C57BL/6 mice were used for generation of SUN1 mutant mice by CRISPR/Cas-mediated genome engineering. Mutant mice were congenic with the C57BL/6 background. Testes and ovaries from mice at different ages as indicated in the manuscript were used for this study.

Wild animals

No wild animals were used in the study.

Field-collected samples

No field collected samples were used in the study.

Ethics oversight

All procedures involving mice and experimental protocols were approved by the regional ethical committee of the National Center for Protein Science Shanghai (approval #SIBCB-S342-1510-043).

Note that full information on the approval of the study protocol must also be provided in the manuscript.

Flow Cytometry

Plots

Confirm that:

- [x] The axis labels state the marker and fluorochrome used (e.g. CD4-FITC).
- [x] The axis scales are clearly visible. Include numbers along axes only for bottom left plot of group (a 'group' is an analysis of identical markers).
- [x] All plots are contour plots with outliers or pseudocolor plots.
- [x] A numerical value for number of cells or percentage (with statistics) is provided.

Methodology

Sample preparation	Ecapsulated testes were dissociated by treatment of Collagenase type I followed by treatment of trypsin. Single-cell suspensions were fixed with ethanol and then stained by PI.
Instrument	BD FACSCalibur Flow Cytometer
Software	BD FACS sortware
Cell population abundance	The whole decapsulated testes were used to prepare single-cell suspensions
Gating strategy	Testicular cells were first gated by FSC-A vs SSC-A to eliminate any debris, then gated for singlets by FSC-H vs FSC-A. spermatocytes were then determined from this gated population by PI staining.

- [x] Tick this box to confirm that a figure exemplifying the gating strategy is provided in the Supplementary Information.
